# Microwave-Assisted Solvent Bonding for Polymethyl Methacrylate Microfluidic Device

**DOI:** 10.3390/mi13071131

**Published:** 2022-07-17

**Authors:** Chia-Wen Tsao, Chang-Yen Chang, Po-Yen Chien

**Affiliations:** Department of Mechanical Engineering, National Central University, Taoyuan City 320, Taiwan; dlam591028@g.ncu.edu.tw (C.-Y.C.); billid0928@gmail.com (P.-Y.C.)

**Keywords:** polymer microfluidics, thermoplastic bonding, solvent bonding, microwave heating

## Abstract

This paper demonstrated a microwave-assisted solvent bonding method that uses organic solvent to seal the thermoplastic substrates with microwave assistance. This direct bonding is a simple and straightforward process that starts with solvent application followed by microwave irradiation without the need for expensive facilities or complex procedures. The organic solvent applied at the bonding interface is used in dissolving and dielectric heating of the thermoplastic surfaces to seal the thermoplastic substrates under microwave assistance. We evaluated acetone and ethanol to seal the polymethyl methacrylate (PMMA) microfluidic device. The bonding performance, such as bonding coverage, geometry stability, and bonding strength (tensile) were observed and compared with the oven-heating and non-heating control experiments under the same force applications. Results showed that the microwave-assisted solvent bonding method presents a high bonding yield (maximum > 99%) and bonding strength (maximum ~2.77 MPa) without microchannel distortion, which can be used for various microfluidic applications.

## 1. Introduction

Microfluidics, initiated from microsystem technology, can handle samples or reagents in a microchannel. Within decades of investigation, microfluidics has devolved into a multidisciplinary technology that incorporates various areas such as chemistry, biology, mechanical engineering, materials science, and bioengineering and has been applied to a wide range of applications [1,2]. In addition to the high detection sensitivity/selectivity and high-level integration, recent investigations have been devoted to developing a more economic, robust, reliable and easy-to-use analytical platform that fulfill the ASSURED (affordable, sensitive, specific, user-friendly, rapid and robust, equipment-free, and deliverable to end-users) requirements for point-of-care testing (POCT) devices, as suggested by the World Health Organization [3]. This will not only help the low-source setting countries to rapidly establish diagnostic capacity but can also improve the diagnostic efficiency and reduce the detection cost for an optimized treatment in developed countries [4,5].

Among various materials used in microfluidics, polymer is a low-cost material with good optical, mechanical, and chemical properties, which is ideal to be used in POCT applications [6,7]. In particular, thermoplastic is a rigid, reliable, and reusable material [8] that can also be amendable to mass-production techniques to produce the microfluidic device in large quantities at a low cost [9]. Fabrication of thermoplastic microfluidics generally involves a front-end microchannel fabrication and a back-end bonding and sealing procedure. [10] While there are numerous thermoplastic replication techniques such as hot embossing [11], injection molding [12], or roller imprinting [13] for the microchannel fabrication, the post-end bonding process is still the bottleneck in terms of fabrication throughput and yield; therefore, post-end thermoplastic bonding is required to be developed. 

The bonding of thermoplastic microfluidic devices has been intensively reviewed and generally classified as direct and indirect approaches [14,15,16]. Adhesive bonding [17] that uses additional glue/epoxy layers as external material to seal the microchannel is categorized as indirect bonding. Methods that directly use thermal [18], mechanical [19], acoustic [20], or microwave energies [21] to seal the thermoplastic substrates are classified as direct bonding. Among all the bonding methods, thermal fusion bonding is one of the most commonly used approaches since it is a simple and straightforward direct bonding approach without using additional materials at the bonding interface. Fusion bonding methods usually involve using a hot embosser [22,23] or convection oven [24] to heat the thermoplastic above the glass transition temperature (T_g_) to seal the device. This results in the excessive heating of the entire substrate, which causes microchannel collapse. Moreover, the aforementioned process usually requires a long time since polymer is a material with low thermal conductivity.

Several advanced interfacial heating methods have been proposed to solve the aforementioned problems. For example, heat can be applied to the bonding interface through ultrasonic acoustic vibrations [20,25,26], laser welding [27,28], and microwave irradiation [21,29,30]. Although these local heating approaches can effectively reduce the excess heating of the entire device, these methods still present their own constrain and limitations. For ultrasonic bonding, partial or excessive fusion occurs due to uneven bonding energy distribution; thus, energy director design is required. For laser welding, an opaque substrate or a conductive metal (titanium) layer is required to absorb the laser for welding. Microwave bonding requires coating a conductive polyaniline or gold layer at the bonding interface to absorb the microwave energy for heating. Among these interfacial heating approaches, microwave is a simple and cost-effective method. Heating can be done by using a regular cheap household microwave oven. Thus, we propose using microwave for directly heating the organic solvent at the bonding interface for microfluidic device bonding.

Solvent bonding is a widely used method for thermoplastic bonding. It differs from fusion bonding in that it mainly relies on thermal diffusion to entangle the polymer chains at the bonding interface, while solvent bonding uses organic solvent to dissolve the polymer surface for bonding [31,32]. Thus, solvent bonding can be mediated by solvent composition, time, and temperature to control the solvated layer for bonding. In addition, to bond the same material, such as PMMA–PMMA [33,34], with proper solvent selection, heterogeneous bonding of different thermoplastic materials such as PLA–PMMA [35], COC–PMMA [36], PMMA/PC, and PMMA/PET [37] can be completed. In solvent bonding, one of the fundamental challenges is excessively softened plastic squeezing into the microchannel, resulting in channel blockage, but with appropriate process control, the clogging issue can be prevented [34]. 

To achieve higher bonding strength and bonding coverage, evaluating the bonding temperature can effectively enhance a more intimate surface contact and increase the polymer chain movement for stronger bonds. However, this will also increase the microchannel clogging problems in the bonding process. To address this concern, the clogging issues can be solved by proper solvent selection; for example, Lee et al. recently demonstrated using acetic acid [38,39] for microchannel clog-free bonding. The bonding can be improved by surface modifications [40] or UV irradiation [38] to reduce the bonding temperature (to room temperature) and enhance the bonding strength. Some special solvent application designs, such as using sacrificial channel [41], solvent imprinting [42], capillary action [43], soak method [44], spin-coating [37], and retention groove [45] have also been invented to prevent an excessive solvent application or polymer reflow into the microchannel. However, these methods require a special microchannel design or particular application method, which limits the fabrication capability and throughput.

In this paper, we demonstrate a microwave-assisted solvent bonding that directly seals the PMMA substrates. Organic solvent is directly applied at the bonding interface for bonding. With microwave irradiation, the organic solvent layer at the bonding interface absorbs the microwave energy and assists heating. This microwave-assisted solvent bonding is a low-cost, simple and straightforward method that doesn’t require expensive high-end facilities to bond the PMMA microfluidic device.

## 2. Experiment

### 2.1. Materials and Reagent

We purchased 2-mm-thick optical-grade (CM-205X) polymethyl methacrylate (PMMA) with a glass transition temperature of 105 °C from CHIMEI Corp. (Tainan, Taiwan). The PMMA glass transition temperature was 105 °C. A two-flute end mill with a diameter of 300 µm was purchased from Taiwan Microdrill Co., Ltd. (New Taipei, Taiwan). A 1.5-mm-diameter stainless steel microdrill was purchased from Taiwan Microdrill Co., Ltd. (New Taipei, Taiwan). Polydimethylsiloxane (PDMS, Sylgard 184 Silicone Elastomer Kit) was purchased from Dow Corning Corp. (Midland, MI, USA). HPLC grade water and Acetone were both purchased from Duksan Techopia Co., Ltd. (Ansan-si, South Korea). Ethanol (EtOH, 99.8%, electronic grade) was purchased from J.T. Baker Chemical Company (Phillipsburg, NJ, USA), and 20 GA × 1/2” Luer Stubs were purchased from Instech Laboratories, Inc. (PA, USA).

### 2.2. Microchannel Fabrication

**Figure 1:**
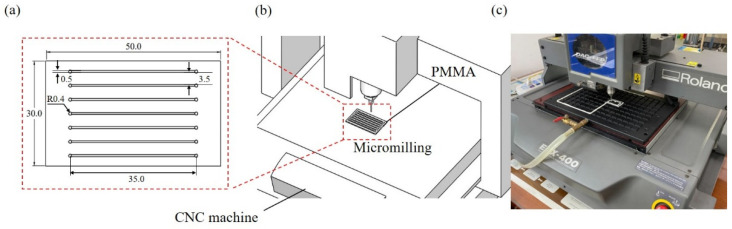
Microchannel fabrication by micromilling. (**a**) parallel microchannel design. (**b**) schematic of desktop router. (**c**) image of desktop router.

As displayed in Figure 1, microchannels were fabricated through computer numerical control (CNC) micromilling to evaluate the bonding performance. PMMA was selected as a major substrate in this study because it is low cost and has good machinability for micromilling. A 2-mm-thick PMMA substrate with an area of 30 × 50 mm^2^ was vacuum-attached to a desktop router (Roland EGX-400, Roland DGA Corporation, Irvine CA, USA) and milled at a spin speed of 8000 rpm and a feed rate of 5 mm/sec to fabricate seven 35 mm (long) × 500 µm (width) × 400 µm (deep) microchannels on the substrate. The inlet and outlet reservoirs can be drilled by a CNC miller with microchannels. In our case, after milling, we drilled the 1.5-mm-diameter reservoirs using a drill press for convenience. Finally, the PMMA substrate was cleaned in an ultrasonic bath, and nitrogen-gun blow drying was conducted to remove polymer particles and debris from the PMMA substrate.

### 2.3. Contact Angle Measurement

The wettability of the PMMA surface before and after surface modification was characterized using an automatic contact angle measurement system (OCA 15EC, DataPhysics Instruments GmbH, Filderstadt, Germany). In each measurement, 4 μL of organic solvents was pipetted onto a 30 × 50-mm^2^ PMMA surface to capture a droplet image and calculate the contact angle by using the automatic measurement system (SCA software for OCA, DataPhysics Instruments GmbH, Filderstadt, Germany).

### 2.4. Bonding Strength Measurements

**Figure 2:**
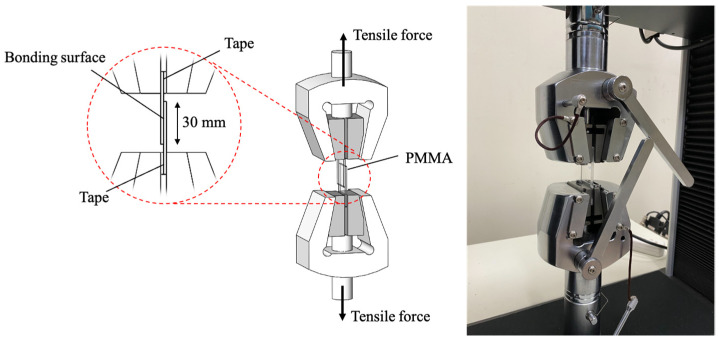
Experiment setup of tensile strength measurement.

The bonding strength was characterized by tensile strength. To determine the tensile strength, a PMMA sample was clamped to a tensile testing machine (HT2402, Hung Ta Instrument Co., Ltd., Taichung, Taiwan), as shown in Figure 2. The sample was pulled at a speed of 0.2 mm/min, and the tensile strength was measured. We recorded the maximum tensile strength when the sample was separated.

## 3. Results and Discussion

### 3.1. Microwave-Assisted Solvent Bonding

In microwave-assisted solvent bonding, the organic solvent is not only used for bonding the PMMA substrates, but also serves as an energy receptor to absorb the microwave irradiation and dielectric heat of the bonding interface due to its good dielectric constant. The choice of solvent and its concentration are the critical factors that fundamentally affect the bonding performance. As displayed in Table 1, acetone and ethanol were selected as major organic solvents in microwave-assisted solvent bonding due to their Hildebrand solubility parameters (acetone *δ*: 20.4, ethanol *δ*: 26.0) being close to that of PMMA substrate (*δ*: 20.1) since the organic solvent and solute (PMMA) can easily coexist and dissolve while the parameters are similar. In addition, they also present a high dielectric constant (acetone ε_s_: 20.7, ethanol ε_s_: 24.5) to absorb microwave energy at the bonding interface. Therefore, we selected acetone and ethanol as our major organic solvent and diluted with water into 25~100% organic solvent concentrations to evaluate the microwave-assisted solvent bonding performance in this research.

**Figure 3:**
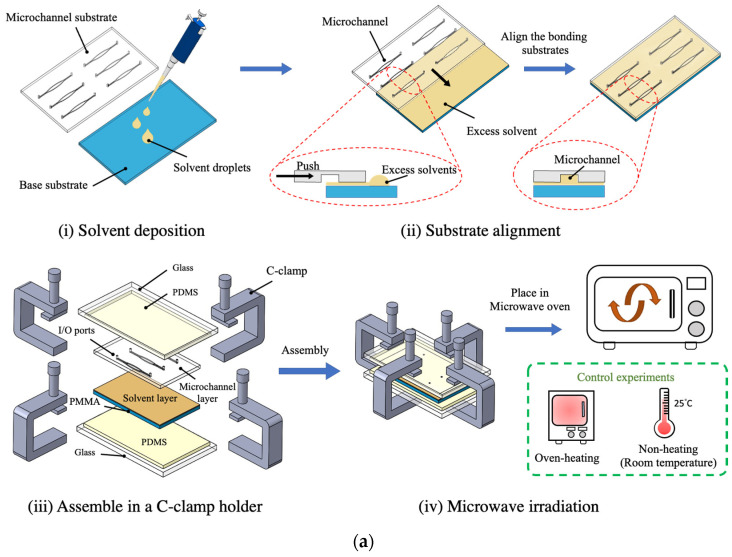

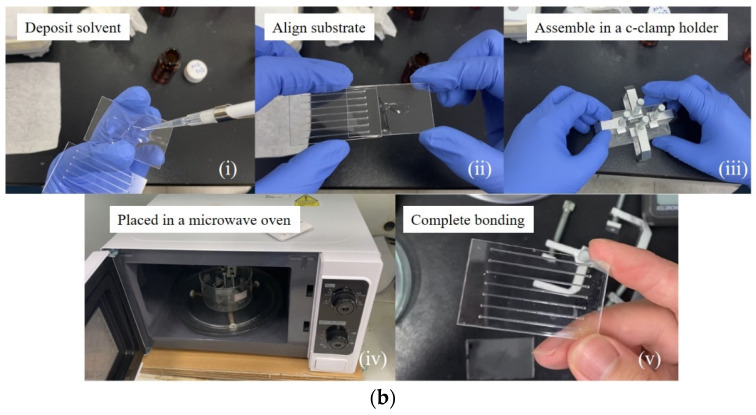
(**a**) Schematic illustration of microwave-assisted solvent bonding procedures, and (**b**) sequential images of bonding procedure.

As shown in Figure 3, the microwave-assisted solvent bonding process starts by uniformly depositing the organic solvents (approximately 100~150 μL in total volume) on the PMMA surface (Figure 3(a-i)). Then, we aligned the substrate and removed excessive organic solvent from the bonding pair (Figure 3(a-ii)). After manual alignment, the whole bonding pairs are stacked with PDMS, and glass, then fastened by C-clamps (Figure 3(a-iii)). The PDMS (or rubber pad) serves as an elastic pad to ensure uniform force application from C-clamp for better bonding uniformity. This bonding assembly was placed in a water-filled glass beaker to prevent the metal clamp arcing during microwave irradiation. Other rigid polymer materials, such as polyetheretherketone (PEEK) or polytetrafluoroethylene (PTFE), could be used as an alternative clamp holder to prevent arcing. However, these polymer holders need to be custom-made. In our approach, in order to have easy tool accessibility, we used standard metal C-clamps that can be purchased from machine shops. After the assembly step, the entire bonding set was placed into the microwave oven (Toshiba MM-MM20P, 700W, Tokyo, Japan) for microwave heating, then cooled down to room temperature (Figure 3(a-iv)). In addition to microwave heating, we also performed solvent bonding with oven-heating and non-heating as control experiments to compare the dielectric heating effects in microwave-assisted solvent bonding. For oven-heating, the entire bonding setup was placed in a drying oven at 65 °C where the PMMA surface heating temperature and time are identical to the microwave-assisting bonding. For the non-heating experiment, the bonding setup was placed at room temperature. C-clamps were also used to hold the bonding pair for both oven-heating and non-heating experiments for force application, which was identical to the microwave heating and oven-heat conditions. The time sequent images of the microwave-assisted solvent bonding process are displayed in Figure 3b.

### 3.2. Microwave-Assisted Solvent Bonding Mechanism

**Figure 4:**
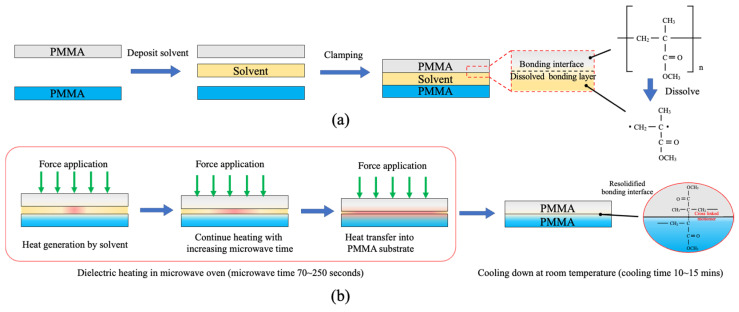
Schematic illustration of microwave-assisted solvent bonding by (**a**) solvent activation and (**b**) microwave irradiation mechanism.

The bonding mechanism of microwave-assisted solvent bonding can be explained by solvent activation and microwave heating mechanism. As displayed in Figure 4a, after solvent deposition, the organic solvent dissolves and diffuses into the PMMA substrates through the case-II diffusion mechanism at the bonding interface [46]. With force application by the C-clamp, the polymer chains are entangled and bond the PMMA surfaces. For the microwave irradiation procedure, through the dielectric heating mechanism [47], the organic solvent absorbs the microwave energy and selectively heats the organic solvent at the bonding interface (Figure 4b). This localized heating by microwave irradiation procedure could also effectively accelerates organic solvent diffusion and form bonds by physical polymer chain interlocking. With microwave irradiation, the temperature in the water beaker was also elevated to ~70 °C, which also assisted in heating the bonding assembly. During the heating and cooling procedure, the organic solvent evaporates out of the PMMA microfluidic device either through bonding interface edges or through microchannel inlet/outlet ports. Finally, the polymer re-solidifies and seals the PMMA substrates.

**Figure 5:**
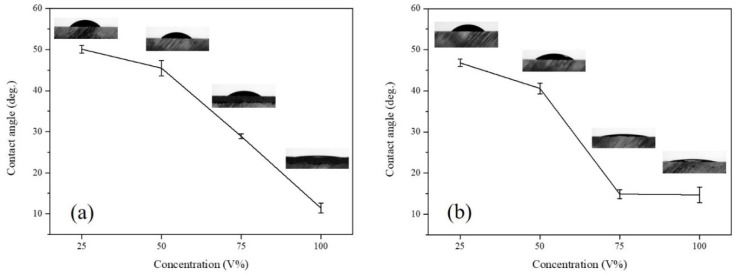
Contact angle measurements of 25~100%; (**a**) acetone, (**b**) ethanol concentrations of PMMA surface.

In microwave-assisted solvent bonding, the liquid-phase acetone or ethanol was directly applied to the PMMA substrate. In the solvent application step (Figure 3(a-ii,b-ii)), excessive organic solvent may fill into the microchannel due to capillary action and therefore needs to be evaluated. Figure 5 displays the organic solvent wettability measurements on the PMMA surface at different concentrations prior to microwave irradiation. For both acetone and ethanol, the contact angle (CA) decreased with increasing organic solvent concentrations. The CA decreased from 50.1 ± 0.9° (25%) to 11.4 ± 1.2° (100%) for acetone (Figure 5a) and for ethanol, as shown in Figure 5b, the CA decreased from 46.8 ± 0.9° (25%) to 14.7 ± 1.9° (100%). This suggests higher organic solvent concentration presents higher capillary pressure, which makes the organic solvent more likely to reside inside the microchannel or causes enhanced wetting of the microchannel surfaces. This may result in microchannel clogging or deformation after bonding.

### 3.3. Microwave-Assisted Solvent Bonding Performance

For bonding performance, we evaluated bonding coverage, geometry stability, and tensile strength with 25~100% acetone and ethanol concentrations on a PMMA substrate with microchannels. The bonding coverage percentage was defined by the effective bonding area divided by the total bonding area. Bonding coverage, geometry stability, and tensile strength results were compared with oven-heating and non-heating conditions with C-clamp force application, which was identical to the microwave-assisted solvent bonding.

#### 3.3.1. Bonding Coverage Evaluation

**Figure 6:**
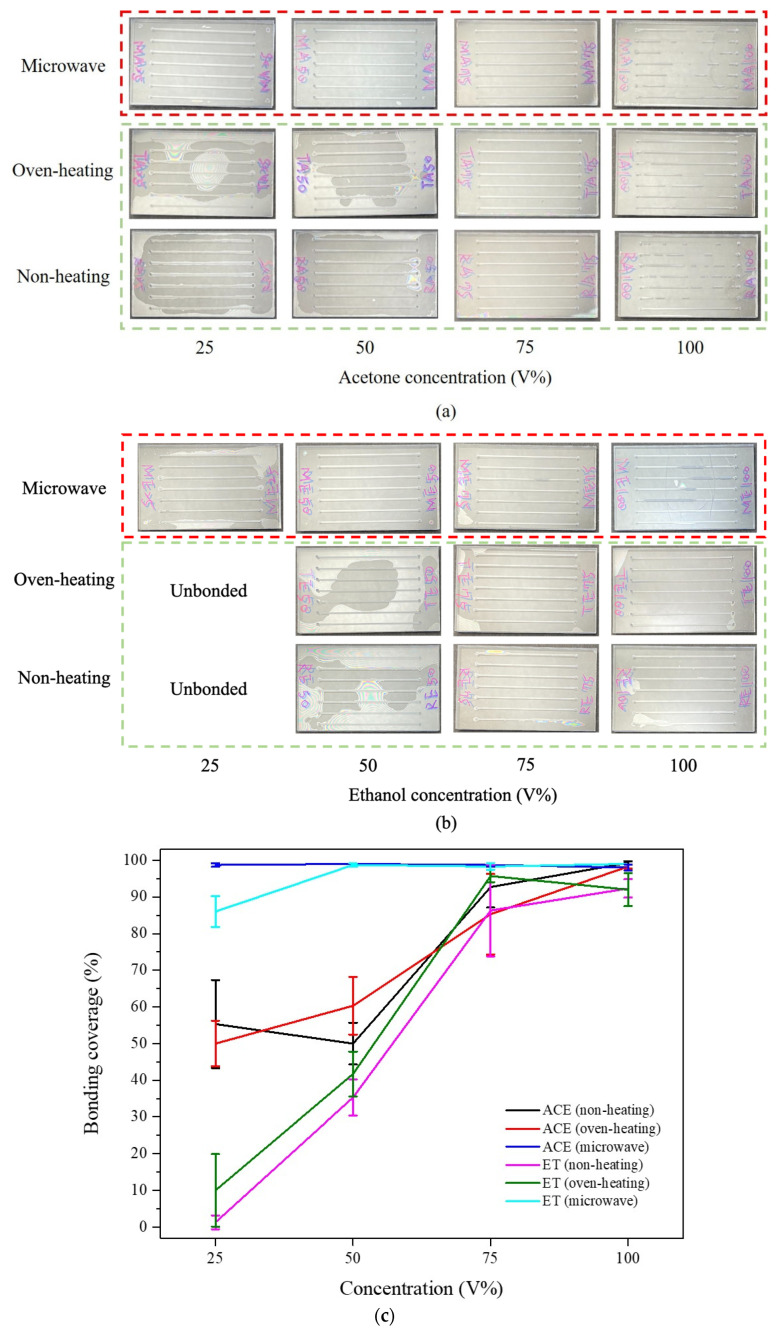
Bonding coverage image of PMMA microchannels with different annealing methods and concentrations for: (**a**) acetone, and (**b**) ethanol. (**c**) summarizes the bonding coverages with different acetone and ethanol concentrations. The error bars in the figure are obtained from more than 3 individual experiments. The microscope images shown in (**a**) and (**b**) were taken by an inverted microscope (Nikon Eclipse Ti, Nikon Corp. Tokyo, Japan). Microwave radiation time was 130 s.

For bonding coverage, as presented in Figure 6, it can be clearly observed that by using microwave-assisted solvent bonding, all the microchannels can be effectively sealed using acetone (red-dash box in Figure 6a). High bonding coverage was obtained even under low organic solvent concentration conditions. Whereas, for the oven-heating and non-heating conditions (green-dash box in Figure 6a), due to insufficient bonding temperature at the interface, low bonding coverages were observed, especially at 25~50% low concentrations. Similar results were also discovered in the ethanol bonding condition (Figure 6b). Figure 6c summarizes the bonding coverage rate with various bonding conditions; ethanol exhibits the lowest bonding rates under either non-heating and oven-heating. At 25% ethanol concentration, the PMMA substrate’s bonding coverage was low (about 1~10%), while acetone demonstrated better bonding coverage than ethanol due to the higher solubility to the PMMA substrate, but still presented low bonding coverage of about 50~55% at 25% acetone concentration. With microwave-assistance, the bonding coverage can reach up to 99% for acetone and 86% for ethanol. These aforementioned results proved that the microwave-assisted solvent bonding process exhibits high thermal heating efficiency for solvent bonding. In contrast, organic solvents are directly heated at the bonding interface through microwave irradiation instead of heating the entire substrate.

#### 3.3.2. Geometry Stability Evaluation

**Figure 7:**
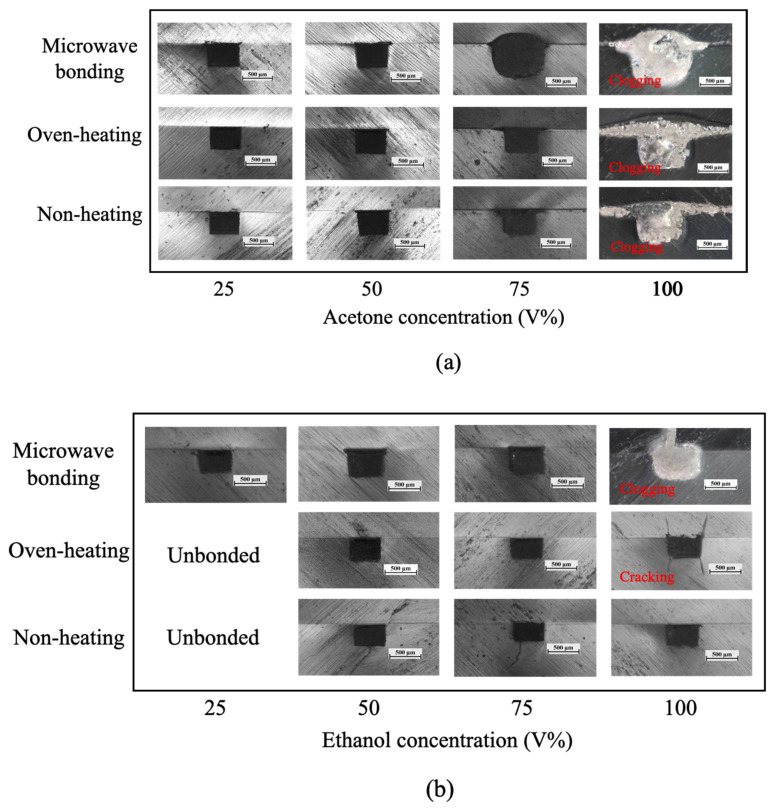
Cross-sectional images of PMMA microchannel with different (**a**) acetone, and (**b**) ethanol concentrations.

Next, we observed the PMMA microchannel cross-section images to evaluate the geometry stability after bonding. As shown in Figure 7, we observed the microchannel cross-section profile change or clogging at high organic solvent concentrations. Since we aim to a have a simple and straightforward bonding process, the organic solvent was directly deposited on the PMMA surface without using a purging step or sacrificial microchannel to remove excess organic solvent from the microchannel. In this case, the organic solvent may be trapped inside the microchannel. Especially for a high organic solvent concentration, which has higher surface wettability (as discussed in Figure 4) the organic solvents are more likely to reside inside the microchannel and be absorbed by the microchannel resulting in microchannel swelling. Therefore, microchannel expansion, clogging, or cracking were observed at 75~100% acetone and ethanol concentration.

#### 3.3.3. Bonding Strength Evaluation

**Figure 8:**
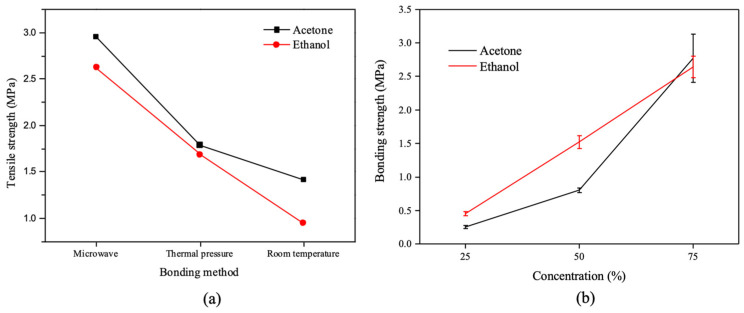
(**a**) Bonding strength measurements for microwave, oven-heating, and non-heating conditions (75% concentration), and (**b**) shows bonding strength measurements with different organic solvent concentrations ranging from 25~75%. The error bars in the figure were obtained from more than 3 individual experiments.

The bonding strength (tensile) measurements are presented in Figure 8. For the microwave-assisted solvent bonding, microwave irradiation helps the organic solvent to directly heat the PMMA surface. As the results show in Figure 8a, microwave-assisted bonding can achieve better bond strength than oven-heating and non-heating conditions. The effects of organic solvent concentration on the bonding strength for microwave-assisted solvent bonding were also evaluated. As shown in Figure 8b, higher organic solvent concentration exhibited a higher bonding strength: with 75% acetone or ethanol concentration, a high bond strength of 2.77 MPa and 2.64 Mpa, respectively, was achieved without microchannel clogging or cracking issues under 100% organic solvent concentration.

**Figure 9:**
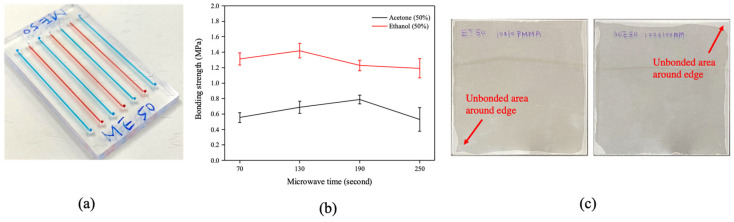
(**a**) Multiplex parrel microfluidic device bonded by a microwave-assisted solvent bonding process (chip size: 3 × 5 cm). (**b**) The bonding strength with different microwave irradiation times from 70 to 250 s. The error bars in the figure were obtained from more than 3 individual experiments. (**c**) Large (10 × 10 cm) PMMA substrate bonding with ethanol (left) and acetone (right). Both ethanol and acetone concentrations were 50%, and microwave irradiation time was 130 s.

Figure 9a displays a multiplex parallel microfluidic device bonded by the microwave-assisted solvent bonding. An optimized 50% organic solvent concentration was selected for both ethanol and acetone, since it presents both high bonding coverage and high bonding strength without microchannel clogging or expansion effects (Figure 7). Besides, bonding PMMA substrates with high solvent concentrations may cause chip cracking. This phenomenon has been previously reported by Rahbar et al. [22]. While these cracks were observed around the microchannel or inlet/outlet ports, they did not cause leakage or fluidic operation and only affected the visual appearance. In our experiments, we found a similar crack phenomenon at 75% and 100% organic solvent conditions, but no cracks were observed in 50% organic solvent concentration. Therefore, we select 50% organic solvent concentration for better chip stability for long-term operation.

The microwave irradiation effects, as shown in Figure 9b, exhibited minor effects on the bonding strength for both ethanol (red line) and acetone (black line), which was presumably due to small amount of organic solvent that resides at the bonding interface. Therefore, a moderate organic solvent concentration of 50% and microwave irradiation time of 130 s was selected for the chip fabrication. Finally, in addition to the regular microfluidic chip size (as shown in Figure 9a), we also tested large area (10 × 10 cm) substrate bonding. As displayed in Figure 9c, high bonding coverages of 94% (ethanol, left image) and 92% (acetone, right image) were obtained. The bonding coverage was slightly less than in the regular condition, which was due to the unbonded area on the substrate edges.

## 4. Conclusions

In this paper, we demonstrated that microwave-assisted solvent bonding is a highly efficient bonding method for sealing microfluidic devices. We evaluated both acetone and ethanol to bond the PMMA substrates with microwave assistance based on the solvent activation and dielectric heating mechanism in microwave-assisted solvent bonding. Solvent bonding and localized heating occur at the same time in the bonding interface. Therefore, higher coverage (maximum > 99%) and higher bond strength (maximum ~2.77 MPa) were obtained by comparing to thermal fusion (oven-heating) or simply solvent bonding (non-heating) conditions; besides, microwave-assisted bonding is a simple, low-cost, and rapid process. The entire microwave-assisted solvent bonding process takes less than 15 min for solvent application, bonding pair assembly, and microwave irradiation. This process only requires a glass beaker, C-clamps, and a <100 USD low-cost conventional household microwave oven to bond the device. With proper organic solvent selection and process control, this bonding method can be applied to other thermoplastic materials (such as cyclic olefin copolymer or polycarbonate). We believe this high-performance microwave-assisted bonding method can be applied in various microfluidic applications with high bonding strength and yield.

## Figures and Tables

**Table 1:** Solubility and dielectric constant of PMMA and organic solvents [14].

Thermoplastic/Solvent	Hildebrand Solubility Parameter, δ [(J/cm^3^)^1/2^]	Dielectric Constant, ε_s_
Polymethylmethacrylate (PMMA)	20.1	4.9
Acetone	20.4	20.7
Ethanol	26.0	24.5
Water	47.7	80.4
